# Professional approaches in clinical judgements among senior and junior doctors: implications for medical education

**DOI:** 10.1186/1472-6920-9-25

**Published:** 2009-05-21

**Authors:** Maria Skyvell Nilsson, Ewa Pilhammar

**Affiliations:** 1 University of Gothenburg, The Sahlgrenska Academy, Institute of Health and Care Sciences, Box 457, SE-405 30 Göteborg, Sweden

## Abstract

**Background:**

Clinical experience has traditionally been highly valued in medical education and clinical healthcare. On account of its multi-faceted nature, clinical experience is mostly difficult to articulate, and is mainly expressed in clinical situations as professional approaches. Due to retirement, hospitals in Scandinavia will soon face a substantial decrease in the number of senior specialist doctors, and it has been discussed whether healthcare will suffer an immense loss of experienced-based knowledge when this senior group leaves the organization. Both senior specialists and junior colleagues are often involved in clinical education, but the way in which these two groups vary in professional approaches and contributions to clinical education has not been so well described. Cognitive psychology has contributed to the understanding of how experience may influence professional approaches, but such studies have not included the effect of differences in position and responsibilities that junior and senior doctors hold in clinical healthcare. In the light of the discussion above, it is essential to describe the professional approaches of senior doctors in relation to those of their junior colleagues. This study therefore aims to describe and compare the professional approaches of junior and senior doctors when making clinical judgements.

**Methods:**

Critical incident technique was used in interviews with nine senior doctors and nine junior doctors in internal medicine. The interviews were subjected to qualitative content analysis.

**Result:**

Senior and junior doctors expressed a variety of professional approaches in clinical judgement as follows: use of theoretical knowledge, use of prior experience of cases and courses of events, use of ethical and moral values, meeting and communicating with the patient, focusing on available information, relying on their own ability, getting support and guidance from others and being directed by the organization.

**Conclusion:**

The most prominent varieties of professional approaches were seen in use of knowledge and work-related experience. Senior doctors know how the organization has worked in the past and have acquired techniques with respect to long-term decisions and their consequences. Junior doctors, on the other hand, have developed techniques and expertise for making decisions based on a restricted amount of information, in relation to patients' wellbeing as well as organizational opportunities and constraints. This study contributes to medical education by elucidating the variation in professional approaches among junior and senior doctors, which can be used as a basis for discussion about clinical judgement, in both pre-clinical and clinical education. Further research is required to explain how these professional approaches are expressed and used in clinical education.

## Background

This study describes senior and junior doctors' professional approaches in clinical judgements. Clinical experience has traditionally been highly valued in medical education and clinical healthcare. It is multi-faceted, consisting of elements such as theoretical knowledge, clinical experience, practical skill and personal maturity [1,2]. Such knowledge is mostly difficult to articulate, and is mainly expressed in clinical situations as professional approaches, including behaviour as well as attitudes.

In Scandinavia, approximately 40% of the medical specialists are more than 55 years of age, and 65% are older than 50. Consequently, there will be a large group of medical specialists reaching retirement age between 2010 and 2020 [3]. These specialist doctors possess a considerable amount of clinical experience, and concerns have been raised concerning whether the healthcare organization will suffer a great loss of experienced-based knowledge that will have consequences for education as well as patient care, when these specialists leave. There have also been discussions about opportunities for doctors to remain in clinical practice after they have reached the legal retirement age [3], which in Sweden is 65 years.

Both senior and junior doctors are traditionally involved in clinical medical education [4]. Studies have shown that doctors are conscious of being a model for junior colleagues and students in clinical practice [4,5], and that role-modelling is also deliberately used in medical teaching [6]. In using such pedagogical models, the clinical educators' professional approaches have a significant impact on the content in focus and the knowledge conveyed to students. Consequently doctors' professional approaches could be expected to have educational consequences.

Cognitive psychology has contributed to our understanding of how clinical experience may influence professional approaches in clinical judgements. The picture that emerges is that experts express different patterns of reasoning compared with those expressed by novices or intermediates, and organize their knowledge differently [7-11]. When doctors get more clinical experience of a situation, their way of reasoning changes from a theoretical/declarative starting-point to one that is based more on experience [8,10,11]. Concepts such as *cues*, *pattern recognition, schemas *and *scripts *have been used to explain and describe the mental structures that more experienced doctors express in clinical judgements [7-10]. Schmidt and Rikers [12] proposed that the development of expertise progresses through a number of transitory stages characterized by qualitatively different expressions of knowledge structures underlying diagnostic performance. On the other hand, other psychologists claim that with more experience, the information process changes from a theoretical instrumental one to a more intuitive one [13]. To understand the difference in professional approaches [14,15], the Dreyfus & Dreyfus model [16] has been used. This model has shown that a person usually passes through five stages of different perceptions of their task, as their skill improves. *The novice *uses context-free facts and rules. *The advanced beginner *considers more objective facts and uses more sophisticated rules. *The competent *performer uses a plan, or chooses a perspective, which then determines which elements of the situation are to be treated as important and which ones can be ignored. *The proficient *performer can see the goal and the important features of the situation but must still decide what action to take. To do this, he or she falls back on detached, rule-based determination of actions. *The expert *not only knows what needs to be achieved but also knows how to realize the goal. Experts reflect on the goal or perspective that seems evident to them, and on the action that seems appropriate to achieve that goal [17].

Additionally, doctors' field of work and their authority often change with greater clinical experience, and they are expected to take more responsibility compared with junior colleagues. Senior doctors are also more often specialized, and meet a group of patients allocated according to specific medical problems. Junior doctors, on the other hand, have their job assignments on the wards and emergency units, where they meet a broad range of cases and problems [18]. How such differences in working position and working responsibility affect doctors' approaches is not so well described in the literature. Consequently we expect doctors' professional approaches to be influenced by clinical experience as well as position and responsibilities.

For these reasons, it is of interest to describe the professional approaches of senior specialist doctors, in relation to those of their junior colleagues. In this study, we focus on examples of clinical judgements to describe professional approaches among senior and junior doctors.

## Methods

### Study design

This research adopted an inductive qualitative approach, and the study was conducted in a department of internal medicine at one of the biggest teaching hospitals in northern Europe, located in Sweden, during 2004.

### Study participants

According to the aim of the study, a purposeful sample of residents (junior doctors) and specialists with considerable clinical experience (senior doctors) was selected. A list including junior doctors (JDs) employed at the internal medicine department (n = 15) was received from the director of studies in the resident programme, and a list presenting senior doctors (SDs) (n = 12) was obtained from the head of the medical school. Presumptive respondents were then selected by the research group in order to acquire broad variation and representation from the speciality of internal medicine (endocrinology, pulmonary diseases, renal diseases, haematology and liver diseases). The proportion of female and male participants was selected to represent the allocation within each age-group population in the region. Selected doctors were informed both verbally and in writing, and consent was obtained before the time of the interview was decided. Two junior doctors declined to participate.

One female and eight male senior doctors (SDs) (aged 65 to 70 years) participated. They were titled professor or associate professor, had more than 30 years of clinical experience and were specialists in their own field of internal medicine. Their clinical work mainly consisted of conducting ward rounds, consultancy and teaching.

Three female and six male junior doctors (JDs) (aged 30 to 44 years) participated. They had between 1 and 10 years of experience as clinical doctors. Five JDs were residents, following a specialist programme in internal medicine (in Sweden, 5 years), and four of them were graduate specialists. All JDs served on a ward and they were all scheduled for on-call service at their clinic.

### Data collection

Eighteen interviews (9 + 9) were conducted by two researchers (MSN and AK). All physicians were interviewed at their clinic, using the critical incident interviewer technique [19], which encourages the interviewed persons to use their own words and concepts when describing self-chosen incidents, experienced in clinical settings. Critical incident technique has previously been used to elucidate important incidents in professions [19] and to elucidate experienced-based knowledge among professionals [20,21]. In this study the critical incident interview technique was used in order to obtain narratives that could illustrate the doctors' professional approaches in clinical judgements. The doctors were asked to recall and describe events (incidents) that had happened during the last year and were experienced as either positively or negatively related to their professional action. The interviews were tape-recorded and transcribed verbatim.

### Ethical Considerations

Permission to carry out the study was given by the head of each department. Informed consent was obtained from all participants in accordance with the Declaration of Helsinki [22]after they were informed of the purpose, method and publication of the study, that participation was voluntary, and that they could withdraw at any time. When this study was planned and conducted, no approval by an ethics committee was required for this type of study according to Swedish law.

### Data analysis

The overall aim of the analysis was to describe the participants' professional approaches, and a qualitative content analysis [23,24] was used to classify their utterances. The analysis was conducted in two phases and several steps:

#### Phase A

The purpose of this phase was to describe the approaches used by both junior and senior doctors in clinical judgements.

1. Meaning units were identified according to the purpose of the study – i.e. clinical judgements were identified. Clinical judgement, in this study, represents statements concerning interpretation or conclusion of a clinical patient's situation that affect the decision to act (or not to act).

2. Each meaning unit was given a code that described the content. Through coding, the researcher becomes familiar with the data and starts to organize the information [23].

3. Meaning units and attached codes were then placed in categories according to the content; for example, *using theoretical knowledge*. These analytical steps are illustrated in Additional file 1. The analysis resulted in eight different categories describing the doctors' professional approaches in clinical judgements, shown in Additional file 2.

#### Phase B

4. Text, meaning units and codes in each category were read in order to detect differences and similarities among junior and senior doctors' approaches. The most prominent, most frequent, and the first mentioned, was detected and described in each category and group. Variations between the two groups were described. Even if this description seems linear it is important to stress that the process of qualitative analysis entails that the researcher goes backwards and forwards between the whole and parts of the text material, during the analysis.

## Results

In the results of this study we describe the way in which junior and senior doctors' professional approaches vary in each category. Quotations to illustrate these variations are presented in the text.

### Using theoretical knowledge

*Theoretical knowledge *(about how to handle a clinical situation/case, based on procedural and scientific knowledge, means-ends rationality) was not prominent or commonly described by SDs. Rather, they expressed theoretical knowledge as a component that is matched against their own experience of different cases and clinical courses. The utterance below illustrates how SDs express theoretical knowledge when diagnosing a patient with suspected instable angina.

There was this worried, Swedish-speaking woman with exactly the same problem [as a man with diffuse symptoms described by the doctor earlier,] /.../...and say that there is no indication of chest pain in the history, for instance, heart enzymes were negative, no on-line recording, and it was quite similar to the other case I talked about. (SD)

In contrast, theoretical knowledge was often used by JDs and seemed to contribute in a substantial way to forming valid clinical judgements. The quotation below illustrates how a JD used theoretical knowledge to reach a conclusion step by step.

Well, that she gets a pain in her chest when she exerts herself, and that nitro that she's been given or Suscard (nitroglycerine) helps, and also, in connection with chest pains, she's fainted several times and that this is very alarming, of course. (JD)

### Using previous experience of cases and courses of events

SDs frequently referred to their experience of previous cases and clinical courses in their clinical judgements. When they said that something was typical, divergent or common, it was in the light of their considerable experience of cases and events. SDs describe how they use prior experience in judgements concerning risks, prognosis, management, decisions concerning difficult and complex situations, and when they interpret examination findings. The quotations below describe how previous experience is used, in recognizing a particular disease/symptom, and in supporting a patient and his/her relatives during a difficult terminal stage.

..... the voice, I said. No, no one had thought anything about that. Strange, I said, so we went up to him and said hello to him, and it was a completely classic example, that this man had a myxoedema. You could tell by his voice, and I hadn't seen the patient, and the pieces all fell into place. (SD)

....I was able to go back and say: I've seen this before, and I know that this is what often happens, you see. Sometimes it goes like this, but sometimes it goes like that...(SD)

JDs had more limited experience, which they did not yet trust fully in clinical judgements. They used their experience of previous cases and events to reflect on their clinical judgements.

Well, it wasn't a Cushing like you see in the books/.../No, you know they are ... the typical ones have thin arms and abdominal fat, and a thick neck and round faces, but she was a bit like that, but/.../I noticed it before but I thought it was so little. (JD)

The type of course that JDs discussed was short, and the statement below illustrates the reasoning concerning the outcome of cardiac arrest situations experienced at the emergency unit.

I mean if you've seen any cardiac arrests in Emergency you realise first of all that not many people make it from Emergency up to the hospital. (JD)

### Adopting an ethical and moral approach

The ethical and moral considerations of SDs were prominent and conclusive, and they referred to their clinical experience in motivating judgements regarding: the approach to the patient, prognostic outcome, physiological and physical consequences as regards the patient's wellbeing, and the risk of "over-treating" and harming patients. The quotations below describe ethical and moral considerations, concerning choice of treatment in relation to effect and the patient's quality of life.

... you shouldn't start using a drug that destroys their life or destroys their quality of life. (SD)

..it's a lot about making sure that such patients are not left for a long time in the respirator, because it's very painful, you know. We place them in the respirator when we judge that we, it's likely that we can get them out after short-term treatment, you see. Otherwise we don't do it because it's so extremely painful. (SD)

JDs' ethical and moral standpoints were mostly seen in their communication with patients and in relation to limited healthcare resources, i.e. using them in the most cost-effective way. Their judgements were mostly founded on their own personal assumption (not experienced-based, as with SDs) of how you should behave in a general ethical and moral manner.

I feel that he isn't capable of really taking advantage of the benefit an operation should give, and you can't simply operate on everybody who has angina, and then we have to choose the ones that have the best chances of benefiting from the result in the best way. (JD)

Well, as far as I'm concerned, I don't think it matters very much, but for the relatives I think it can be very helpful [to see their dead relative before the respirator is disconnected]. (JD)

### Meeting and communicating with the patient

SDs drew attention to the unique elements in each meeting with patients and situations. They also emphasized the importance of communication in clinical work and underlined the patients' vulnerability and needs. SDs also described how they handled their own needs and controlled their behaviour when communicating with the patient.

I don't show that I'm irritated, angry, pressed for time, or if the patient is insolent. I try to take it in good part, because I can. (SD)

Their description reflected two-way communication, as exemplified in the statements below.

... this woman, she was a gifted woman who understood her situation well and wanted, it was obvious, she wanted to have information about how she should cope [with a difficult course of illness] and her husband wanted that too. (SD)

Well, it's a way [talking with the patient] of getting the patients to take their medicines, to make them understand, of course they must be informed about why they have their medicines and why they need to take them, otherwise they might skip them. (SD)

JDs based their approach to patients on general clinical procedures and focused their attention on how to act, and on providing information. The statements below show that one- way communication was typical of the doctor-patient relationship described here.

..it's always important to take a history, and I did, of course, here, and I was going to take that type of history, I was going to, I did take it. (JD)

It's very important to go in and say hello to the patient ./.../It's the first contact, so that the patient knows who he or she is talking to. So then you can say, like, I'm a specialist or a house officer, because they know I'm not a consultant. So they can't demand answers to all the questions, and sometimes I can't, so to speak. (JD)

### Focusing on available information

SDs' statements showed that they searched for and used available information in, for example, medical records. This approach was considered to facilitate an early diagnostic hypothesis and economize with healthcare resources.

You can kind of skim through quickly and still see things without really searching for them, but if you practise it, it works reasonably well in any case, and then I found an answer that wasn't recorded in the discharge notes, that he had a digitalis [dose] of around 4, which is definitely toxic, isn't it, and problems with digitalis when [due to] hypersensitivity, and then it's.... then you haven't made use of the information either, have you? (SD)

JDs described situations where they had to make judgements based on limited information, due to insufficient/or lack of time, medical records, medical examination results and/or experience/knowledge. This implies that judgements had to be made according to the information and time available.

There are twenty-eight patients lying and waiting for you and the corridors are full, and then you make a decision, as I mentioned a bit before, and the whole job consists of decisions, of course. Is this a clot, no it isn't a clot. Well, what is it then? And then you decide something, and then you've made that decision. (JD)

### Relying on one's own ability

SDs were aware of their role as experts in a field. They knew how to act and knew what to do in complicated cases. They also knew they were the professionals who had to make the final judgement. Personal qualities such as honesty, integrity and self-knowledge were suggested as being important characteristics, for facilitating high-quality judgements and in relations with patients.

Knowledge and it's, I mean, the strength a specialist has in an area, it's that you can a bit more safely say that now it's not possible to do any more. There's nothing more that can be done and you know that then, and feel and say that this is a correct judgement, and the patients feel that too, you see. That's the way it is, you see. Perhaps it's an important component in the whole situation. (SD)

Being honest with yourself and with the patients makes it easier to handle these difficult things, both lack of knowledge and when we can't do anything more. (SD)

JDs also had to trust their ability to act in the right way, and had to be able to explain their reasons for action, though their confidence in their own ability sometimes seemed limited. The statements indicate that this seemed to be due to the fact that their role as a doctor was not fully developed or that they lacked clinical experience.

So I thought, from my amateurish point of view, from that perspective, I thought it would be best to operate on him, but ... I can feel that this is not something for me to decide. (JD)

They could also experience limitations in ability, for reasons connected with age and sex.

... he's been in and tried to talk to the patient too, because it can be a good thing sometimes. In other words, a man and a bit older, then they might understand, because I mean I look more or less like the assistant nurses or registered nurses, you see. (JD)

### Getting support and guidance from others

All the same, SDs knew they were experts, and they turned to other experts to get confirmation on clinical judgements in order to create greater trust in patients. They also turned to other personnel with considerable clinical experience, for opinions about judgements.

...or a patient comes and says oh, I had such a pain in my chest all night, and then you see a strange ECG and then I sometimes run to a colleague with a bit more experience and discuss an ECG, and then I tell the patient that I've talked to a heart specialist here, and then they look rather pleased. (SD)

They're confined to bed for a long time after transplantations and sometimes it affects the problem of managing the operation itself, so that the physiotherapist also plays a certain part in assessing muscle function after the transplantation. Often they're the ones who've decided or think that this (situation) is too bad. (SD)

JDs frequently asked for support and opinions from others when making clinical judgements. There were four main reasons for doing this: to consult colleagues within their own speciality; to hand over responsibility; to obtain specialist knowledge other than their own; and to obtain another doctor's opinion on a clinical judgement that they had made. JDs also compared their own clinical judgements with those reached by other more experienced doctors. They were greatly influenced by judgements made by more experienced doctors and they described how they followed their advice despite having doubts.

...interpretations and tests are also very important, and it can also be tricky, and then you ring and wait for the doctor. They don't have to be much older either, so long as they have worked longer than I have. (JD)

I had relied completely on what psych [the psychiatric specialist] had advised about medication. (JD)

### Being directed by the organization

JDs expressed that they were directed by the healthcare organization when making clinical judgements. There were restrictions due, for example, to shortage of staff, lack of time and shortage of hospital beds.

I usually go and check in the old records if I don't have access to the current one, and I hadn't got it just then. It was about six in the morning and they couldn't get it out then. (JD)

Organizational directions were also expressed as opportunities in the healthcare organization; for example, as knowledge about specialist care (experts and specialist wards) and procedures in managing patient care (investigation procedures). In the statement below, the JD expressed such directions in taking into consideration when the patient was to have her X-ray, and decided that the patient would have to remain in hospital.

There's probably a one- to two-week wait for this coronary artery X-ray, so she has to keep a place here. (JD)

In this category there were no statements from SDs. This could be related to the fact that senior and junior doctors have different working conditions, with regard to both hours and duties. Another explanation could be that these situations were very familiar to senior doctors and were therefore not mentioned.

## Discussion

The aim of this study was to describe professional approaches in clinical judgements made by senior and junior doctors. Most studies describing differences in clinical judgements between doctors with varying degrees of experience have almost exclusively focused on diagnostic performance [25,26]. In this study, the participants described situations that they discerned in their professional work, which allowed a more comprehensive description of the professional approaches in clinical judgements. Although our investigation was limited to a selected number of senior and junior doctors at one hospital and one speciality in medicine, it has resulted in important findings concerning the way clinical experience influences the use of knowledge and focus in clinical situations.

SDs' statements indicate that their experience was expressed in a job that was dominated by making judgements concerning diagnoses and treatments in a restricted medical speciality. The observed predominant use of prior experience of cases and courses of events among SDs is consistent with the findings of studies on expert performance [14,27], and their statements indicate that they use cues [8], patterns [9] and make associations, with considerable clinical experience of cases and situations with patients [28]. Their clinical experience seems to have a prominent role in all aspects of clinical judgements, including ethical and moral judgements. Their statements indicate great clinical proficiency; they seem to know the clinical procedures and what can be excluded in their field. SDs focus on what is unique and specific in the situations described. This is in agreement with Benner's description of expertise [14] (building on the skill model of Dreyfus & Dreyfus [16]). "As the skill model predicts, with more experience comes a better grasp of the nature of particular clinical situations, including opportunities and constraints. Consequently responses to patients become more contextualized and attuned. Recognition of clinical situations moves from abstract textbook accounts of general features to an experience-based response to the situations" [14] (pg 190). SDs also expressed a wider view of clinical judgements, which implies that they focus on long-term decisions, and that they have enough experience to know what to do to reach their goal, and what to exclude. They expressed examples of a (more) differential and developed ethical and relational skill [14], and their way of reasoning is in accordance with the description of expertise [16].

JDs' clinical approaches, on the other hand, were based on varied levels of skill; i.e. on whether they were advanced beginners, competent, and had become proficient or experts [16]. According to Benner [21], developing from novice to expert occurs mainly through experience of a particular activity over a period of time, and the findings indicate that JDs describe both new and more familiar clinical situations. JDs do not express the wider views that senior doctors do, and their focus is on the process and their ability to act. In this study, theories derived from cognitive psychology do not serve as the best explanations concerning JDs' judgements. The model of skill acquisition suggested by Dreyfus and Dreyfus [16] reflects the professional approaches described by JDs more distinctly and comprehensively. Most of the judgements expressed by the JDs were based on the skill level of an advanced beginner or someone who is competent, characterized by the use of guiding rules or informal yardsticks learned from past experience with other patients, by actively searching for credible sources of good and useful information (practice of colleagues), and by being anxious to perform without making mistakes [14].

According to Benner [21], most qualitatively distinct differences in the professional skill model lie between the competent and proficient levels, where the practitioner begins to read the situation. There are some examples where JDs express judgements that can be associated with a proficient skill level [14], e.g. when they use previous experience and courses of events (they focus on the narratives). A practical grasp of the situation is expressed, which reflects the skill of seeing practical manifestations of changes in physiological states, patients' responses, and of noticing these transitions [14]. In such cases, JDs sought someone with more experience or chose to follow safe and tested rules and routines that they were familiar with.

Besides making judgements about diagnoses and treatment, the JDs focused on finding the best and most suitable track for patients to follow through the healthcare organization, with respect to the patients' wellbeing and the organizational conditions. Their statements also indicate that they have developed expert knowledge concerning these clinical problems. Here they show an ability to combine the content of the rules into a whole, relate it to the meaning of the context and then act accordingly [21]. Judgements directed by the organization were not expressed by SDs, and we suggest that this finding reflects different working conditions and responsibilities in the healthcare organization, where JDs have developed a competence, seeking to find a balance between professional demands in relation to organizational opportunities and limitations. These differences between junior and senior doctors can also be seen as differences in interpreting the problem. Such differences have previously been described, where house staff appear to reorganize the facts according to potentially clarifying testing procedures, while experts (specialists) reorganize facts into clusters corresponding to the causal relationships in the disease schemata [10].

An illustration of the most prominent variations in clinical approaches, stated in clinical judgements, among junior and senior doctors, is given in Additional file 3.

### Methodological considerations

The strengths of this study include the use of critical incident interview technique [19], which has previously been used to elucidate experienced-based knowledge among professionals [20,21,29]. It is also verified that information collected with CIT is both reliable and valid [30]. The interviews were performed in a place chosen by the informants, at there own clinic. The researchers were not connected with the informants' workplace and the informants were not in any way dependent on the researcher. This probably contributed to the fact that they could speak very openly about their experience; they were also given a lot of time without being interrupted while speaking. All of the informants spoke Swedish and were thoroughly informed about the aim of the study. A supportive relationship between the interviewer and participants was emphasized, as it is very important in CIT interviews where the intention is to elicit negative and positive events in healthcare [31]. In order to create accuracy of the technique, the interviewer's role was to encourage precise and accurate behavioural descriptions, and to help informants to be as specific as possible in their description of a particular incident [19]. This requires a trained interviewer to be successful [31]. Flanagan [19] also underlines the significance of the interviewer's knowledge concerning the subject in focus. The interviewers, in this study, had previous experience both as interviewers and in research concerning experienced knowledge, as well as considerable clinical experience of healthcare.

Content analysis is a research method that uses a set of procedures to make valid inferences from text and involves defining the content to be studied, the concepts to be measured, the unit of analysis, the sampling plan, and the scheme for categorizing the content [23].

The research groups (MSN and EPA) participated in each analytical step, and their interpretation is supported by quotations from participants. The extent to which these findings are due to generational differences or the proportions of men and women in the study groups has not been in focus here, but will be presented more closely in a forthcoming investigation (Skyvell Nilsson M, Pennbrant S, Samuelsson B, Pilhammar E: Professional attitudes mediated in clinical education: an ethnographic study, submitted). The limitations of this study are the number of participants and the restriction to one speciality in medicine and to one hospital. Hence, the generalization of the findings is limited. It should be emphasized that the findings are clearly based on the doctors' statements, and in our opinion other researchers would only be able to point to minor differences.

## Conclusion

The result shows how professional experience and knowledge is expressed in clinical judgements. The result indicates that each generational group develops unique knowledge and experience that is based on social, organizational and personal prerequisites and experience. Senior doctors know how the organization has worked in the past and have acquired techniques with respect to long-term decisions and their consequences; they use more experience-based knowledge in clinical judgements. Junior doctors, on the other hand, have developed techniques and expertise for making decisions based on a restricted amount of information, in relation to patients' wellbeing as well as organizational opportunities and constraints; they use more declarative and theoretical knowledge in clinical judgements. Consequently we suggest that both groups could contribute to clinical education. However, the expressions and differences in professional approaches among senior and junior educators have to be elucidated and explored in order to benefit students' understanding of professional development. Consequently, we imply that this study contributes to medical education in at least four ways:

1. It creates an understanding of how senior and junior doctors differ in their use of knowledge in clinical situations. Such knowledge is crucial in order to understand how professional experience is constituted and developed. The perceptual insight, or intuition, that experienced senior doctors have developed could make it difficult for them to relate to the struggles of early learners (novices). On the other hand, these experienced doctors could act as role models in order to show how perceptual knowledge is deliberately used in clinical judgements.

2. It elucidates the knowledge used in clinical situations by both junior and senior doctors, and could thus be helpful in ensuring that the knowledge conveyed in theoretical classes corresponds to the knowledge required in clinical situations. The introduction of such knowledge early in medical education might assist students in the transition between pre-clinical and clinical training in medical education.

3. It facilitates for reflective learning. By clarifying the professional approaches used by both junior and senior doctors, these approaches could be put forward and scrutinized by both teachers and learners, in order to facilitate for further professional development.

4. It is helpful to student and clinical educators in clarifying the differences between specialist expertise (senior doctors) and generalist expertise (junior doctors).

Finally, this study describes how junior and senior doctors differ in their use of knowledge and focus. Further research is required to reveal how these differences are expressed in clinical education, for example by exploring the effect of professional approaches to the way in which supervision is performed, and by studying the focus of content in clinical education.

## Competing interests

The authors declare that they have no competing interests.

## Authors' contributions

MSN and EP have contributed to the design of the study. MSN and AK carried out the interviews. MSN wrote the first draft of the paper. MSN and EP contributed to the analysis of the data, interpretation of results and the final version of the manuscript.

## Authors' Information

Ewa Pilhammar is Professor of Health Care Pedagogics, with several years of experience of studies relating to experience-based knowledge, and of using qualitative research methods, e.g. the critical incident technique.

Maria Skyvell Nilsson is a doctoral student in Health Care Pedagogics.

Ewa and Maria have been studying doctors' experience-based knowledge since 2004.

## Pre-publication history

The pre-publication history for this paper can be accessed here:



## Supplementary Material

Additional File 1 **Figure 1**. Examples of text analysis, phase A. Click here for file

Additional File 2 **Figure 2**. Categories describing strategies used in clinical judgements. Click here for file

Additional File 3 **Figure 3**. Prominent approaches in clinical judgements among junior and senior doctors. (X means that this approach was prominent.) Click here for file
