# Supplementary material for: Professional approaches in clinical judgements among senior and junior doctors: implications for medical education
Source: BMC Med Educ. 2009 May 21;9:25. doi: 10.1186/1472-6920-9-25 (PMC2693513; doi:10.1186/1472-6920-9-25)
Supplement: Additional File 1 — Figure 1. Examples of text analysis, phase A. [file 1472-6920-9-25-S1.doc]

| **Text** | **Meaning unit** | **Code** | **Category** |
| --- | --- | --- | --- |
| …we have this basic attitude, you know, that if a person enters into an acute dyspnoea due to a temporary infection, with some complication, we don’t give up, but in her case ***the thing was that it wasn’t a temporary deterioration but the end of a long decline,*** and that’s where specialist experience comes in, being able to make a decision that this is how it is. (SD) | ***the thing was that it wasn’t a temporary deterioration but the end of a long decline*** | ***Previous cases and courses of events*** | ***Using previous experience of cases and courses of events*** |
| …then I also think that it’s ***better to spend those resources on someone who can benefit more from them.*** For example, a 40-year-old lying there and dying of a cerebral haemorrhage. (JD) | ***better to spend those resources on someone who can benefit more from them*** | ***Allocating resources*** | ***Adopting an ethical and moral approach*** |
| ..Well, it may be connected with this condition. ***An unusual manifestion, but the same old thing applies, and every patient is unique. Really a patient has his own condition*** and even if it doesn’t seem typical, you have to be ready for any emergency/on the alert in this kind of condition to meet the patients and quickly, and it didn’t sound good, full of anxiety, so… (SD) | ***An unusual manifestion, but the same old thing applies, and every patient is unique.* *Really a patient has his own condition*** | ***Seeing the uniqueness*** | ***Meeting and communicating with the patient*** |
| ..so he made it. He lay there, see, and gasped for breath so that no, yes that was it. ***And then I rang the lung department and got hold of a good lung doctor,*** and he thought it was the ventilation, because that was what I felt that it was – a ventilation problem – and so we discussed it. (JD) | ***And then I rang the lung department and got hold of a good lung doctor*** | ***Using an expert*** | ***Getting support and guidance from others*** |
