# Supplementary material for: Professional approaches in clinical judgements among senior and junior doctors: implications for medical education
Source: BMC Med Educ. 2009 May 21;9:25. doi: 10.1186/1472-6920-9-25 (PMC2693513; doi:10.1186/1472-6920-9-25)
Supplement: Additional File 2 — Figure 2. Categories describing strategies used in clinical judgements. [file 1472-6920-9-25-S2.doc]

*Using theoretical knowledge*

*Using previous experience of cases and courses of events*

*Adopting an ethical and moral approach*

*Meeting and communicating with the patient*

*Focusing on available information*

*Relying on one’s own ability*

*Getting support and guidance from others*

*Being directed by the organization*
