# Supplementary material for: Professional approaches in clinical judgements among senior and junior doctors: implications for medical education
Source: BMC Med Educ. 2009 May 21;9:25. doi: 10.1186/1472-6920-9-25 (PMC2693513; doi:10.1186/1472-6920-9-25)
Supplement: Additional File 3 — Figure 3. Prominent approaches in clinical judgements among junior and senior doctors. (X means that this approach was prominent.) [file 1472-6920-9-25-S3.doc]

| *Professional approaches in clinical judgements* | *SDs* | *JDs* |
| --- | --- | --- |
|  |  |  |
| *Using previous experience of cases and courses of events* | *X* |  |
| *Adopting an ethical and moral approach* | *X* |  |
| *Meeting and communicating with the patient* | *X* |  |
| *Focusing on available information* | *X* |  |
| *Relying on one’s own ability* | *X* |  |
| *Using theoretical knowledge* |  | *X* |
| *Getting support and guidance from others* |  | *X* |
| *Being directed by the organization* |  | *X* |
